# Expression and characterization of a novel 1,3-regioselective cold-adapted lipase from *Rhizomucor endophyticus* suitable for biodiesel synthesis

**DOI:** 10.1186/s13068-016-0501-6

**Published:** 2016-04-14

**Authors:** Qiaojuan Yan, Xiaojie Duan, Yu Liu, Zhengqiang Jiang, Shaoqing Yang

**Affiliations:** Bioresource Utilization Laboratory, College of Engineering, China Agricultural University, Beijing, 100083 China; College of Food Science and Nutritional Engineering, Beijing Advanced Innovation Center for Food Nutrition and Human Health, China Agricultural University, Beijing, 100083 China

**Keywords:** Lipase, *Rhizomucor endophyticus*, Gene cloning, *Pichia pastoris*, Cold-adapted, Biodiesel synthesis

## Abstract

**Background:**

The biodiesel production can be carried out by transesterification using either chemical or enzymatic process. The enzymatic transesterification is more promising as it offers an environmental friendly option compared to the chemical process, where the lipases with high catalytic efficiency and good stability play a key role. Hence, it is of great value to identify novel lipases which are suitable for biodiesel production.

**Results:**

A lipase gene (*ReLipA*) from *Rhizomucor endophyticus* was cloned and heterologously expressed in *Pichia pastoris*. ReLipA shared the highest identity of 61 % with the lipases from *Rhizopus delemar*, *Rhizopus oryzae*, and *Saccharomyces cerevisiae*. The recombinant lipase (ReLipA) was secreted as an active protein with the highest activity of 1961 U mL^−1^ in a 5-L fermentor by high cell-density fermentation. ReLipA was purified to homogeneity with a recovery yield of 75.7 %. The purified enzyme was most active at pH 6.0 and 40 °C, respectively, and it was stable up to 55 °C. ReLipA displayed 75 % of its maximal activity at 0 °C, indicating that it is a cold-adapted lipase. It exhibited broad substrate specificity toward various *p*-nitrophenyl esters and triglycerides. ReLipA hydrolyzed triolein to release mainly 1,2-diolein without the formation of 1,3-diolein, suggesting that it is a *sn*-1,3 regiospecific lipase. Furthermore, ReLipA synthesized different types of oleates by esterification using oleic acid and short chain alcohols (e.g., methanol, ethanol, and butanol) as the substrates with the highest conversion yield of 82.2 %. Therefore, the cold-adapted lipase may be a good biocatalyst in ester synthesis in biodiesel industry.

**Conclusions:**

A novel cold-adapted lipase was identified and characterized. The high yield and excellent properties may confer the enzyme with great potential for biodiesel production in bioenergy industry. This is the first report on a cold-adapted lipase from *Rhizomucor* species.

## Background

The increasing demand for energy, depletion of fossil fuel reserves, heightened awareness of climate change due to greenhouse gas emissions, and environmental pollution have led to the development of alternative renewable energy sources [1, 2]. Biodiesel, the mixture of alkyl esters, produced by catalytic transesterification of glycerides which are commonly from nonedible or waste oils with short chain alcohols, is a potential alternative to fossil fuels because it is renewable, biodegradable, and nontoxic [2]. The biodiesel production can be carried out by transesterification using either chemical or enzymatic process. The enzymatic transesterification is more promising as it offers advantages with an environmental-friendly option compared to the chemical processes, such as mild reaction condition, less energy intensity, higher yield in esters, as well as better recovery of glycerol and the transesterification glycerides with high free fatty acid contents [3]. Lipases with high catalytic efficiency and good stability play a key role in biodiesel production process.

Cold-active/adapted lipases show high activity at low temperatures in comparison to the lipases from mesophiles or thermophiles, which have their activities drastically reduced at low temperatures [4]. Recently, cold-active/adapted lipases have been found to be attractive in biodiesel production over other lipases mainly in terms of energy saving, since the biodiesel synthesis by most other lipases was performed at elevated temperatures [2, 4–6]. To date, many cold-active/adapted lipases have been identified and characterized, most of which are from bacteria [4, 7–10]. Only few cold-active/adapted lipases have been reported from fungi, such as *Aspergillus nidulans* [11], *Geotrichum* sp. [12], and *Penicillium expansum* [13]. In general, the yields of cold-active/adapted lipases by wild-type strains are very low, and hardly meet the requirements for large-scale production. Encouragingly, these can be overcome by high-level heterologous expression of lipase genes from various microorganisms in suitable hosts. So far, some cold-active/adapted lipases have been gene cloned, expressed, and characterized, such as the lipases from *Geomyces* sp. P7 [14], *Psychrobacter* sp. G [15], *P. cryohalolentis* K5 [9], and *Yersinia enterocolitica* [16], and microbial environmental genomes [17–19]. However, few cold-adapted lipase genes from fungi have been cloned and expressed [14]. Moreover, no gene encoding cold-adapted lipase has been reported from *Rhizomucor* species*. Pichia pastoris* is an attractive host for the cost-efficient production and engineering of heterologous (eukaryotic) enzymes due to several advantages, such as high efficiency and low production cost [20]. To date, several mesophilic lipase-encoding genes have been successfully expressed in *P. pastoris* [20, 21], but only a few cold-adapted lipase genes have been expressed in *P. pastoris* [4]. Besides, the yields of cold-adapted lipases still remain low [22–24], and the highest yield of 2760 U mL^−1^ was observed from CALIP1 which was derived from a metagenomics library [25].

Esters with long-chain fatty acids have long been used as intermediate materials in large quantity for the production of fatty acid derivatives, which have been widely used as biodiesel in fuel industry, food additives in food industry, as well as fragrances in cosmetic industry [26]. Among them, biodiesel mono-alkyl esters produced from oils or fats have properties similar to those of petro-diesel, but burning biodiesel results in lower emissions of particulates, CO, SO_x_, and aromatic hydrocarbons [3]. To date, some lipases have been used to catalyze the synthesis of biodiesel from vegetable oils or waste cooking oils [3, 27–29], but the catalytic efficiency is still not high enough for industrial production, and there is hardly any information on the biodiesel production using cold-adapted lipases [30, 31].

The strains from the genus *Rhizomucor* have been reported to be good producers of lipases and esterases [32, 33]. *Rhizomucor endophyticus* is an endophytic zygomycete species in higher plants that grows well at 18–28 °C [34]. To the best of our knowledge, no lipase from *R. endophyticus* has ever been reported. In this paper, we describe gene cloning, expression, and biochemical characterization of a novel cold-adapted lipase from *R. endophyticus*. The potential of the recombinant lipase in biodiesel synthesis was further evaluated.

## Results

### Cloning of a lipase gene and sequence analysis

**Fig. 1:**
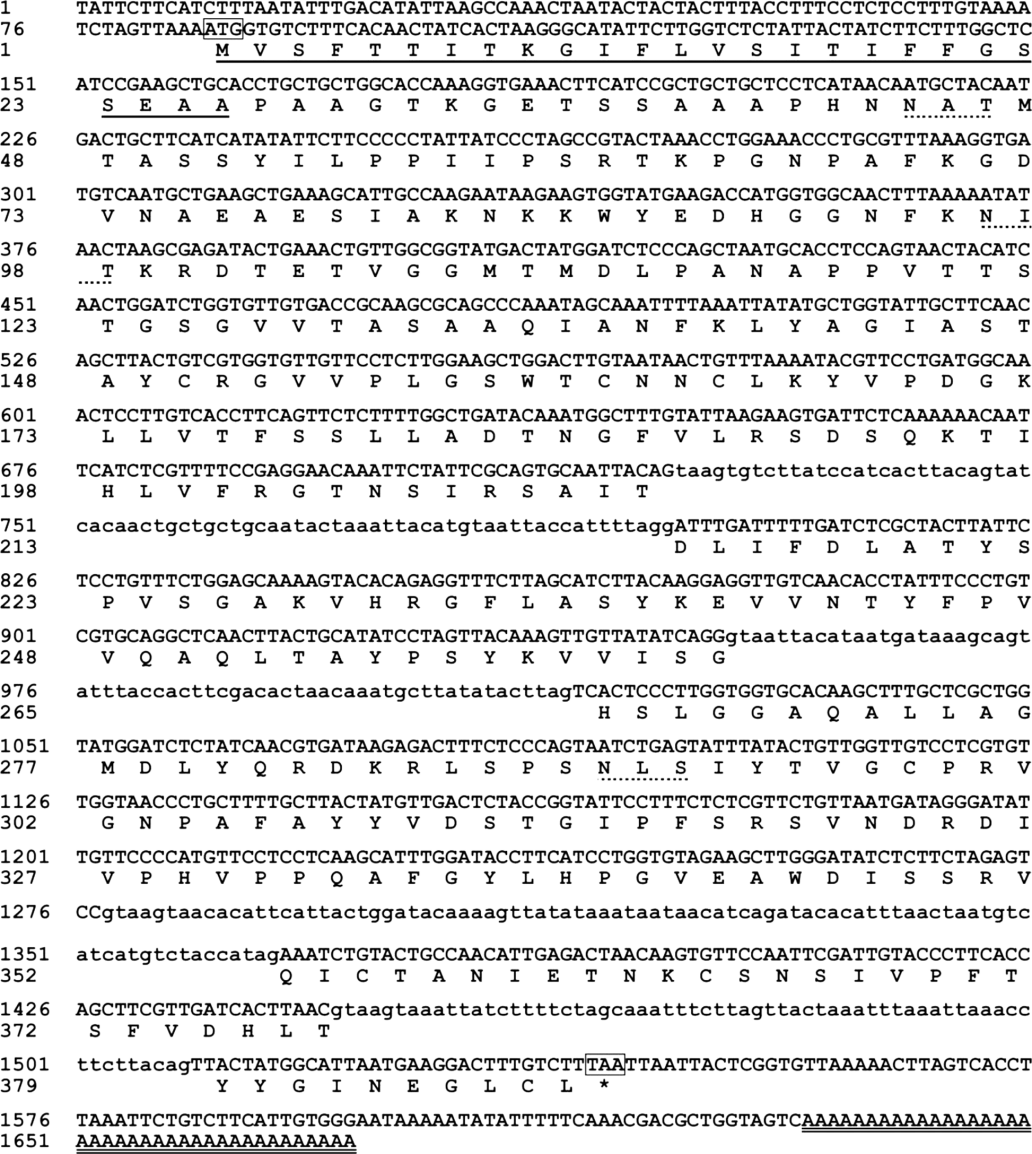
Nucleotide and deduced amino acid sequences of the lipase (ReLipA) from *R. endophyticus*. The translational initiation codon (ATG) and termination codon (TAA) are *boxed*. Intron sequences are shown in *lowercase letters*. A putative signal peptide is indicated *underline*. The *N*-linked glycosylation sites are marked by *dotted line*. A poly (A+) tail is *double underlined*. The nucleotide sequence reported here has been submitted to GenBank under accession number, KF203134

A 210-bp partial gene from *R. endophyticus* was amplified by PCR using the degenerate primers: LipDF and LipDR. Sequence analysis revealed that the amplified fragment had the motif of lipase superfamily. The 5′ and 3′ flanking regions of the fragment amplified by RACE were approximately 1067 and 486 bp, respectively. After assembling the two flanking regions, the full-length lipase cDNA of 1381 bp (*ReLipA*) was obtained with an open reading frame (ORF) of 1167 bp (Fig. 1). There are four introns with lengths of 75, 63, 89, and 64 bp interrupted in the coding region. The mature protein has a molecular mass of 41,464 Da and a theoretical *p*I of 6.07. The protein sequence contained three possible *N*-glycosylation sites. The nucleotide sequence has been deposited in the GenBank under the accession number, KF203134.

**Fig. 2:**
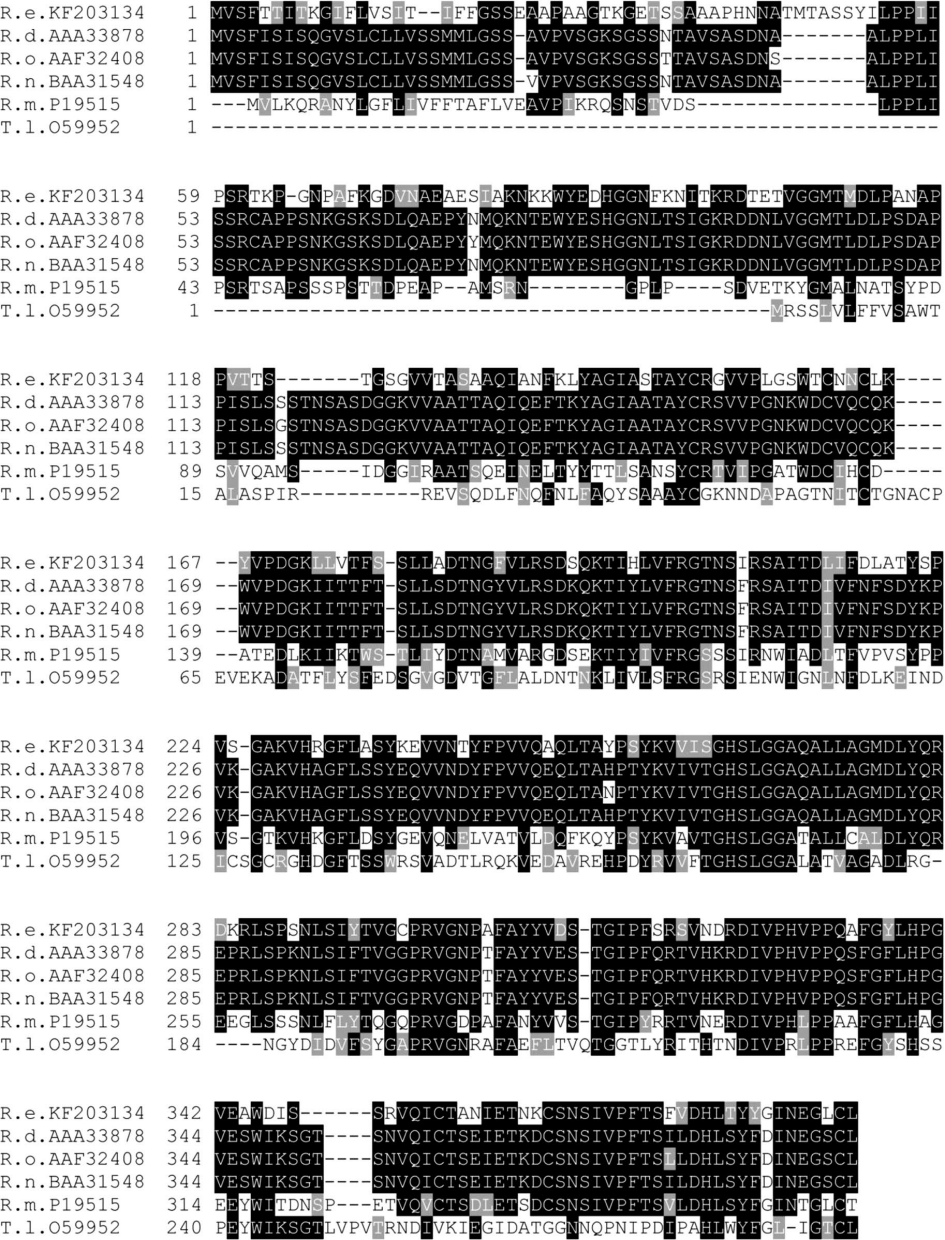
Multiple alignment of amino acid sequences of ReLipA and other several lipases. *Numbers* on the *left* are the residue numbers of the first amino acid in each *line*. Abbreviations and accession numbers of those lipases are as follows: *Rhizomucor endophyticus* (R.e. KF203134), *Rhizopus oryzae* (R.o. AAZ31460), *R. niveus* (R.n. BAA02181), *R. chinensis* (R.c. ABN59381), *R. stolonifer* (R.s. AAZ66864), and *R. delemar* (R.d. EIE75333). Identical residues are shaded in *black*, and conserved residues are shaded in *gray*. The conserved catalytic motif is *underlined*. The putative catalytic nucleophile and acid/base are identified by *filled circle*

Sequence analysis revealed that ReLipA contained a catalytic triad consisting of Ser266, Asp325, and His376 residues, and a well-conserved GHSLG motif in the active site of Ser266. ReLipA shared the highest identity with the characterized lipases from *Rhizopus delemar* (61 % identity, AAA33878), *Rhizopus oryzae* (61 %, AAF32408), and *Saccharomyces cerevisiae* (61 %, BAA31548), followed by the lipase from *Thermomyces* (*Humicola*) *lanuginose* (38 %, O59952) (Fig. 2).

### Expression of *ReLipA* in *P. pastoris* by high cell-density fermentation

**Fig. 3:**
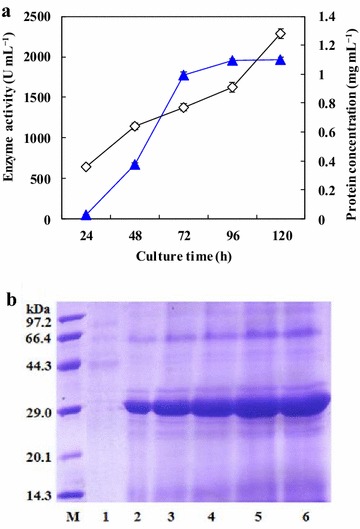
Time-course profile of secretory expression of ReLipA in *P. pastoris* by high cell-density fermentation (**a**), and SDS-PAGE analysis of the secreted proteins during the fermentation process (**b**). (*Filled square*) lipase activity; (*filled circle*) cell wet weight; and (*filled triangle*) protein concentration. *Lane M*, low molecular weight standard proteins; *lane 1* before methanol induction; *lanes 2–6* culture supernatants after 24, 48, 72, 96, and 120 h of induction, respectively

The lipase gene (*ReLipA*) without signal sequence was subcloned into the vector pPIC9 K under the control of the methanol-inducible *P. pastoris AOX1* promoter. The recombinant plasmid was transformed into *P. pastoris*, and the positive transformants were screened. One transformant secreted lipase with the highest activity (15 U mL^−1^) in the shake-flask culture. The strain was subjected to high cell-density fermentation in a 5-L fermentor, and the highest lipase activity of 1961 U mL^−1^ with a protein content of 1.28 g L^−1^ was achieved after 120 h of fermentation (Fig. 3).

### Purification of the recombinant lipase

**Fig. 4:**
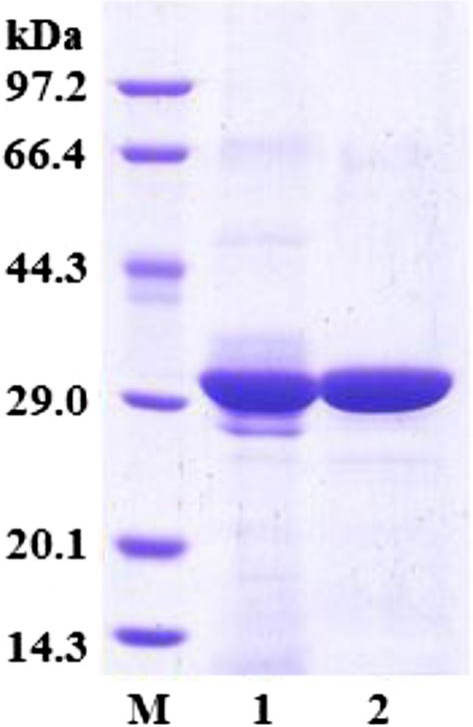
SDS-PAGE analysis of the proteins during the purification process of ReLipA expressed in *P. pastoris*. *Lane M*, low molecular mass protein standards; *lane 1*, crude protein extract; *lane 2*, purified lipase after SP Sepharose column purification

ReLipA was purified to homogeneity with a 1.1-fold purification and a recovery yield of 75.7 % (Table 1). The purified enzyme migrated as a single band on SDS-PAGE with a molecular mass of 33.0 kDa (Fig. 4), while the native molecular mass of ReLipA was determined to be 37.7 kDa, indicating that ReLipA is a monomer.

**Table 1:** Purification summary of the recombinant lipase (ReLipA) from *R. endophyticus* expressed in *P. pastoris*

Purification step	Total activity (U)^a^	Total protein (mg)^b^	Specific activity (U mg^−1^)	Purification (fold)	Yield (%)
Crude enzyme	11711	10.6	1099	1.0	100
SP-Sepharose	8871	7.6	1169	1.1	75.7

^a^Enzyme’s activity was determined in 50 mM citrate buffer (pH 6.0) at 40 °C for 10 min using *p*NPL as the substrate

^b^Protein concentration was measured by the method of Lowry et al. [43] using BSA as the standard

### Characterization of the recombinant lipase

**Fig. 5:**
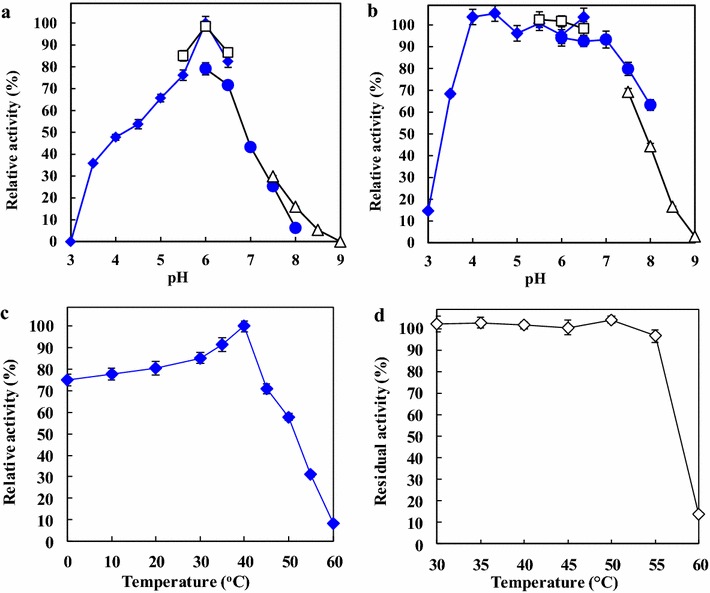
Optimal pH (**a**), pH stability (**b**), optimal temperature (**c**), and thermostability (**d**) of ReLipA. Optimal pH was determined by measuring the enzyme’s activity at 40 °C in 50 mM of different buffers within pH range of 3.0–9.0. The buffers used were citrate buffer (*filled diamond*) (pH 3.0–6.0), MES buffer (*filled square*) (pH 5.5–6.5), phosphate buffer (*filled triangle*) (pH 6.0–8.0), and Tris–HCl (×) (pH 7.5–9.0). To investigate pH stability, the samples were incubated at 30 °C for 30 min in various buffers mentioned above, and the residual activities were then measured in citrate buffer (pH 6.0) at 30 °C. The optimal temperature was determined at different temperatures (0–60 °C) in 50 mM citrate buffer (pH 6.0). For thermostability determination, the enzyme was incubated in 50 mM citrate buffer (pH 6.0) for 30 min at 30–60 °C prior to enzyme assay

ReLipA exhibited a maximal activity at pH 6.0 (Fig. 5a), and was stable within the pH range of 4.0–7.0 (Fig. 5b). The optimal temperature of the lipase was found to be 40 °C, and the enzyme displayed relatively high activities at low temperatures, retaining 75 % of its maximal activity at 0 °C (Fig. 5c). ReLipA was stable up to 55 °C, as more than 90 % of its activity was retained after incubation for 30 min (Fig. 5d).

The influences of some chemicals on lipase activity of RelipA were investigated (data not shown). The lipase activity was significantly enhanced in the presence of Tween 20 (138 %), Tween 60 (157 %), Tween 80 (195 %), and Triton X100 (294 %), while it was completely inhibited by SDS (0 %). In addition, ReLipA was obviously activated by methanol (113 %), cyclohexane (154 %), and heptanes (169 %), but moderately inhibited by butanol (88 %), and strongly inhibited by acetone (31 %), acetonitrile (1.5 %), and isopropanol (0.9 %).

### Substrate specificity, kinetic parameters, and positional specificity of ReLipA

ReLipA preferentially hydrolyzed *p*NP esters with medium chain lengths of fatty acids and exhibited the highest activity of 1611 U mg^−1^ toward *p*NPC (C_8_), followed by *p*NPL (C_12_), *p*NPD (C_10_), and *p*NPH (C_6_) (Table 2). Low specific activity was observed toward *p*NPP (C_16_), and no activity was detected toward *p*NPA (C_2_) (Table 2). Besides, ReLipA showed the highest activity of 1823 U mg^−1^ toward tricaprylin (C_8_) when triglycerides were used as the substrates, and exhibited activities toward the triglycerides with carbon chain lengths of fatty acid in the range of 4–16 (Table 2). The kinetic parameters, *K*_m_ and *V*_max_ values of ReLipA toward *p*NPC, *p*NPL, and *p*NPM were determined to be 2.3 ± 0.21 mM and 1288 ± 48.5 μmol min^−1^ mg^−1^, 0.64 ± 0.06 mM and 384.8 ± 17.5 μmol min^−1^ mg^−1^, and 0.1 mM ± 0.01 and 185.8 ± 7.51 μmol min^−1^ mg^−1^, respectively (Table 3).

**Table 2:** Substrate specificity of the purified lipase (ReLipA) from *R. endophyticus*

Substrate	Chain length	Specific activity (U mg^−1^)	Relative activity (%)
*p*NP esters			
*p*NPA	C_2_	0	0
*p*NPB	C_4_	852	53
*p*NPH	C_6_	941	58
*p*NPC	C_8_	1611	100
*p*NPD	C_10_	1137	71
*p*NPL	C_12_	1297	81
*p*NPM	C_14_	936	58
*p*NPP	C_16_	277	17
Triglycerides			
Triacetin	C_2_	0	0
Tributyrin	C_4_	1164	64
Tricaproin	C_6_	1167	64
Tricaprylin	C_8_	1823	100
Tricaprin	C_10_	715	39
Trilaurin	C_12_	421	23
Trimyristin	C_14_	106	6
Tripalmitin	C_16_	44	2.4

Enzyme’s activity was determined in 50 mM citrate buffer (pH 6.0) at 40 °C for 10 min

**Table 3:** Kinetic parameters of ReLipA

Substrate	*K*_m_ (mM)	*V*_max_ (μmol min^−1^ mg^−1^)	*K*_cat_ (s^−1^)	*k*_cat_/*K*_m_ (mg^−1^ s^−1^)
*p*NPC (C_8_)	2.30 ± 0.21	1288.0 ± 48.5	0.891	0.387
*p*NPL (C_12_)	0.64 ± 0.06	385.3 ± 17.5	0.267	0.416
*p*NPM (C_14_)	0.10 ± 0.01	258.1 ± 10.5	0.179	1.79

The kinetic parameters of ReLipA were determined at 40 °C in 50 mM citrate buffer (pH 6.0) for 5 min using different substrate concentrations

**Fig. 6:**
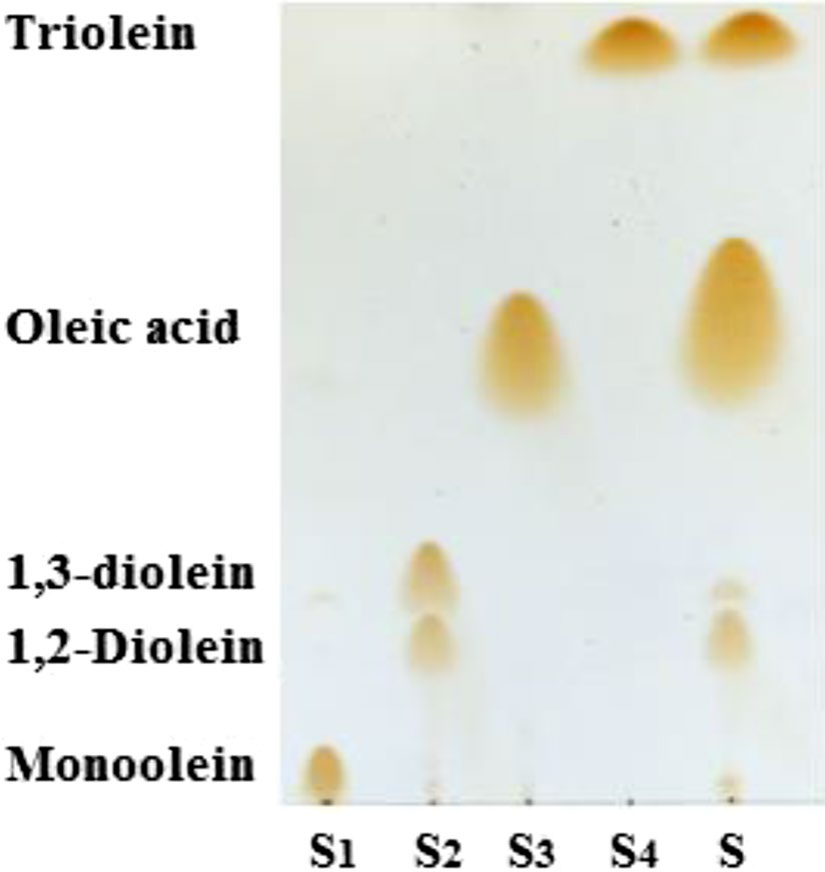
TLC analysis of the hydrolysis products of triolein by ReLipA from *R. endophyticus*. *Lanes S*_*1*_ *–S*_*4*_, standards, *lane S*_*1*_ monoolein; *lane S*_*2*_ diolein; *lane S*_*3*_ oleic acid; *lane S*_*4*_ triolein; *lane S* hydrolysis products

In order to examine the positional specificity of ReLipA, the hydrolysis of triolein was performed. TLC analysis of the hydrolysis products indicated that ReLipA hydrolyzed triolein to release only 1,2-diolein without formation of 1,3-diolein (Fig. 6), suggesting that ReLipA may be a *sn*-1,3 regiospecific lipase.

### Synthesis of butyl oleate

**Fig. 7:**
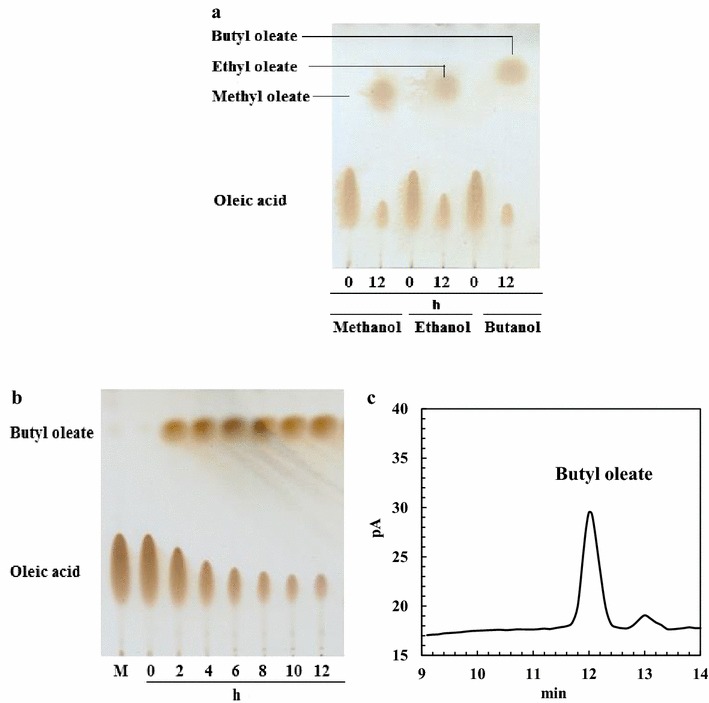
Synthesis of biodiesel from oleic acid and alcohols by ReLipA. (**a**) TLC analysis of the synthesized products. (**b**) Time-course profile of butyl oleate production from oleic acid and butanol. (**c**) Gas chromatography analysis of synthesized butyl oleate after 12 h of incubation. For biodiesel synthesis, 100 U of lipase was added into a mixture containing 1.68 g oleic acid and 1098 μL alcohol (methanol, ethanol, or butanol contained in a 25-mL flask), and incubated at 35 °C for 24 h. Samples taken at different time intervals were qualitatively and quantitatively determined by TLC and gas chromatography

ReLipA catalyzed the synthesis of biodiesel by esterification using oleic acid and alcohols (methanol, ethanol, and butanol) as the substrates (Fig. 7a). After optimization of reaction conditions, the highest conversion ratio of 82.2 % (*w/v*) for butyl oleate production was obtained after 12 h of incubation (Fig. 7b, c).

## Discussion

Lipases have been attracting great attention in recent years due to their wide application fields especially in biodiesel production [35]. So far, a number of microbial lipases have been identified, heterogeneously expressed and characterized, among which only a few are cold-active/adapted lipases [4, 8, 11, 12, 14, 16, 19]. Here, for the first time, we reported gene cloning, high-level expression, and biochemical characterization of a novel cold-adapted lipase (ReLipA) from *R. endophyticus* and its application in biodiesel synthesis.

ReLipA shared medium sequence similarity (61 %) with several characterized lipases from *Rhizopus delemar*, *Rhizopus oryzae* and *Saccharomyces cerevisiae*, indicating that ReLipA may be a novel lipase. Sequence analysis revealed that the amino acid residues viz. Ser266, Asp325 and His376, forming the catalytic triad in fungal lipases are highly conserved, with Ser266 located in a highly conserved pentapeptide motif GHSLG (Fig. 2), further suggesting that the enzyme belongs to Lipase Super family.

The lipase gene (*ReLipA*) was successfully expressed in *P. pastoris*. In order to improve the lipase production, fed batch cultivation was conducted in a 5-L fermentor. The highest lipase activity of 1961 U mL^−1^ was secreted after 5 days of cultivation with methanol feeding induction (Fig. 3). This value is much higher than those of most other cold-active/adapted lipases, including the enzymes from *Yarrowia lipolytica* [22], *Stenotrophomonas maltophilia* [23] and *Microbacyterium luteolum* [24], which only produced 1.5, 2.4, and 8 U mL^−1^, respectively. It is only next to that of a cold-active lipase from a metagenomic library [25]. However, the activity is comparable or obviously lower than those of some mesophilic lipases. For example, Wu et al. [21] have cloned a lipase gene from *Rhizopus chinensis* which was expressed in *P. pastoris*, and the highest lipase activity of 2130 U mL^−1^ was obtained after optimization of induction conditions. Yu et al. [20] expressed a lipase gene (*proARO*) from *Rhizopus oryzae* in *P. pastoris* and improved the lipase yield up to 12019 U mL^−1^ by controlling proper NH_4_^+^ concentration. The molecular mass of ReLipA was estimated to be 33 kDa on SDS-PAGE (Fig. 4), which is similar to that of the cold-active lipase from *P. cryohalolentis* (33 kDa [9]), but lower than those of most other reported cold-active/adapted lipases with molecular masses in the range of 35–65 kDa [6, 14, 15].

ReLipA was most active at a weakly acidic pH (6.0) (Fig. 4a), which is different from most other fungal lipases that generally exhibit optimal activity at neutral or alkaline pH [12, 14, 19]. Although the optimal temperature of ReLipA (40 °C) is higher than that of most other cold-active/adapted lipases which have optimal temperatures in the range of 15–35 °C [4], it exhibited high relative activities at low temperatures, retaining 75 % of its maximum activity even at 0 °C (Fig. 4c). The value is obviously higher than that of the other cold-active/adapted lipases, such as the lipases from *Geomyces* sp. P7 (15 % [14]), *Aspergillus nidulans* (30 % [11]), and *Geotrichum* sp. (58 %, lipase-A [12]). It is only a little lower than that of the cold-active lipases from *Geotrichum* sp. (lipase-B [12]) and *P. cryohalolentis* [9], both of which retained 80 % of their maximal activity at 0 °C. It is noteworthy that ReLipA exhibited a high specific activity of 1208 U mg^−1^ protein at 0 °C, which is much higher than that of most other cold-active/adapted lipases [9, 11, 12, 14]. Moreover, ReLipA showed excellent stability up to 55 °C, which is higher than that of most other reported cold-active/adapted lipases that are stable at temperatures below 50 °C [4]. However, the recombinant cold-adapted lipase from *G.* sp. retained 100 % of its activity after incubation at 100 °C for 1 h [14]. The properties of high specific activity at low temperatures and excellent thermostability may confer the enzyme with great potential in various industrial applications, especially those performed at low temperatures, such as cheese making, dairy production, and biosynthesis of heat-sensitive chemicals.

ReLipA exhibited a broad range of substrate specificity toward *p*NP esters with different carbon chain lengths of fatty acid (4–16) (Table 2). The high specific activity toward *p*NPC (C_8_), *p*NPL (C_12_), and *p*NPD (C_10_) was observed, suggesting that it is a true lipase. The substrate specificity of RelipA is similar to that of most other cold-active/adapted lipases, showing the highest specific activity toward *p*NPC (C_8_ [7, 32]), *p*NPD (C_10_ [17]), and *p*NPL (C_12_ [30]). However, several other cold-active/adapted lipases preferred to hydrolyze *p*NP esters with short carbon chain lengths of fatty acid [12, 15]. In addition, lipases have selectivity to the positions of the ester bonds in triglycerides. Based on the bond selectivity, lipases have been classified into different groups. Most of the lipases fall into *sn*-1,3 regiospecific and non-regiospecific groups; *sn*-1,3 regiospecific lipases act on the ester bonds on positions 1 or 3 in triglycerides, while non-regiospecific lipases act on the three positions randomly. So far, few lipases have been reported to show *sn*-2 selectivity [5]. ReLipA hydrolyzed triolein to yield only 1,2-diolein without formation of 1,3-diolein (Fig. 6), indicating that ReLipA is a *sn*-1,3 regiospecific lipase. The lipase from *Rhizomucor miehei* is also a *sn*-1,3 regiospecific lipase [36]. It has been reported that the lipases with *sn*-1,3 regiospecific property are useful in the production of structured lipids [35, 37]. Therefore, ReLipA may have potential applications in fat and oil modifications.

Esters of long-chain fatty acids are increasingly used as biodiesel in fuel industry. They are traditionally produced using oils and alcohols as the substrates via chemical method, which not only has a low conversion yield and generates some byproducts, but also consumes a large amount of concentrated acids thus leading to environmental pollution [38]. Recently, enzymatic synthesis by lipases has been gradually developed as a promising environmental friendly way for biodiesel production [2, 26]. Despite the mentioned advantages, the application of biocatalysts in biodiesel synthesis has some obstacles, such as low biocatalyst productivity (due to low reaction rate and low stability) and high cost of enzyme [2]. To date, many attempts have been made to develop highly efficient enzymatic process for biodiesel production, especially to identify novel lipases suitable for biodiesel production [2, 39, 40]. For example, Singh et al. [28] isolated a novel lipase from *Schizophyllum commune*, and found that the enzyme was able to produce fatty acid methyl esters from oil and methanol with the highest yield reaching to 94 %, exhibiting potential for application in biodiesel industry; however, the yield of lipase was low. Ayaz et al. [3] identified and characterized a lipase from *Streptomyces* sp., and found that the lipase had transesterification ability using olive oil and methanol as the substrates. Yan et al. [41] developed an integrated process with coupled lipase production and in situ biodiesel synthesis in a recombinant *P. pastoris* yeast, and the highest biodiesel yield reached up to 87 % after the optimization of synthesis conditions. The novel lipase from *R. endophyticus* in the present study exhibited excellent ability to catalyze the synthesis of methyl oleate, ethyl oleate, and butyl oleate in nonaqueous isooctane solvent system with a maximum yield of 82.2 % (Fig. 7). In addition, the enzyme yield is high enough for commercial production, and the enzyme is stable in different organic solvents. All these results indicate that ReLipA may be a good candidate in biodiesel production in biofuel industry.

## Conclusions

A novel cold-adapted lipase (ReLipA) from *R. endophyticus* was gene cloned, heterologously expressed, and biochemically characterized. The highest lipase activity of 1961 U mL^−1^ was produced in a 5-L fermentor by high cell-density fermentation. ReLipA was most active at pH 6.0 and 40 °C. It exhibited a cold-adapted nature, exhibiting 75 % of its maximal activity at 0 °C. ReLipA showed broad substrate specificity and *sn*-1,3 positional selectivity. Besides, the enzyme was suitable for the synthesis of biodiesel. The excellent properties may make the enzyme to be of great potential for wide applications in bioenergy industries.

## Methods

### Strains, plasmids, and reagents

*Escherichia coli* DH5α (Biomed, Beijing, China) was used for propagation of plasmids, and *P. pastoris* GS115 (his4) was used for protein expression. Trizol reagent (Invitrogen, Carlsbad, USA) and Oligotex mRNA Midi Kit (Qiagen, Dusseldorf, Germany) were used for total RNA extraction and mRNA purification. BD SMART™ RACE cDNA Amplification Kit was purchased from Clontech (Palo Alto, CA, USA). Ex Taq DNA polymerase, PrimeSTAR HS DNA polymerase, restriction endonucleases, and pMD18-T were purchased from TaKaRa (Tokyo, Japan). T4 DNA ligase was purchased from New England Biolabs (Ipswich, MA, USA). The *Pichia pastoris* expression kit was obtained from Invitrogen (Carlsbad, USA). *p*-Nitrophenol (*p*NP), *p*NP acetate (*p*NPA), *p*NP butyrate (*p*NPB), *p*NP hexanoate (*p*NPH), *p*NP caprylate (*p*NPC), *p*NP decanoate (*p*NPD), *p*NP laurate (*p*NPL), *p*NP myristate (*p*NPM), and *p*NP palmitate (*p*NPP) were purchased from Sigma Chemical Co. (St. Lous, MO, USA). *p*NP hexanoate (*p*NPH) was obtained from HEOWNS Company (Tianjin, China). Olive oil, soybean oil, and peanut oil were purchased from a local market. All other chemicals used were of analytical grade unless otherwise stated.

### Microorganism and RNA extraction

*Rhizomucor endophyticus* deposited in the China General Microbiological Culture Collection Center (CGMCC) under accession number 3.4684 was used in this study. For isolation of genomic DNA, *R. endophyticus* was inoculated in the medium and cultured at 25 °C for 4 days with a rotation speed of 200 rpm. The medium contained (g L^−1^): soybean 20, yeast extract 10, tryptone 10, MgSO_4_·7H_2_O 0.3, KH_2_PO_4_ 5, and CaCl_2_ 0.3. Fungal mycelia were collected by centrifugation (5000×*g*, 10 min), washed twice with sterilized water at 4 °C and then ground to fine power in liquid nitrogen. The total RNA was extracted with the Trizol reagent, and mRNAs were purified using the Oligotex mRNA Midi Kit.

### Cloning of a lipase gene from *R. endophyticus* and its sequence analysis

The degenerate primers: LipDF (5′-CGGCCACTCCCTGggnggngcnca-3′; n = A/T/C/G) and LipDR (5′-TGAGGAGGGACGTGGggnacdatrtc-3′; n = A/T/C/G, d = A/G/T, r = A/G) were designed on the basis of two conserved sequences (TGHSLGGAQ and RDIVPHVPPQ) of known fungal lipases using the CODEHOP algorithm [42]. PCR was performed using *R. endophyticus* genomic DNA as the template. PCR conditions were as follows: a hot start at 94 °C for 5 min, ten cycles of 94 °C for 30 s, 60–55 °C for 30 s, and 72 °C for 1 min, followed by 20 cycles of 94 °C for 30 s, 55 °C for 30 s, and 72 °C for 1 min. The PCR products were gel-purified, ligated to pMD18-T vector, and sequenced.

The full length cDNA sequence of the lipase was obtained by 5′ and 3′ RACE (rapid amplification of cDNA ends) using a BD SMART™ RACE cDNA Amplification Kit (Clontech, Palo Alto, CA, USA). 5′ end of the cDNA was amplified using the primer Lip5′GSP (5′-AGGAGGAACATGGGGAACAATATC-3′) and adapter primer UPM, followed by a nested PCR using nested gene-specific primer Lip5′NGSP (5′-GAACAATATCCCTATCATTAACAGAAC-3′) and adapter primer NUP. For the 3′ end of the cDNA, the primary PCR was performed with two primers: Lip3′GSP (5′-CACTCCCTTGGTGGTGCACAAGC-3′) and UPM, followed by a nested PCR using Lip3′NGSP (5′-CAAGCTTTGCTCGCTGGTATG-3′) and NUP. The PCR conditions for RACE were: 5 min at 94 °C, followed by 30 cycles of 30 s at 94 °C, 30 s at 62 °C and 1 min at 72 °C, and finally 10 min at 72 °C. The PCR products were gel-purified, ligated to pMD18-T vector, transferred into *E. coli* DH5α for sequencing, and subjected to BLAST analysis.

Sequence assembly and analysis were performed with DNAMAN software (LynnonBiosoft, USA). Database homology searches of nucleotide sequences were carried out using BLAST in GenBank at the NCBI (http://www.blast.ncbi.nlm.nih.gov/Blast.cgi). Multiple alignment analysis was performed by Clustal W program (ftp-igbmc.u-strasbg.fr/pub/ClustalW/). Signal peptide was analyzed at SignalP 4.0 server (http://www.cbs.dtu.dk/services/SignalP/). *N*-Glycosylation sites were predicted using NetNGlyc1.0 (http://www.cbs.dtu.dk/services/NetNGlyc/).

### Expression of the lipase gene in *P. pastoris*

The lipase gene (*ReLipA*) without signal sequence was amplified from the genome of *R. endophyticus* using the primers: ReLipAF (5′-ATTCCGGAATTCCCTGCTGCTGGCACCAA-3′) and ReLipAR (5′-AAATATGCGGCCGCTTAAAGACAAAGTCCTTCATT-3′). Two restriction sites, *EcoR*I and *Not*I were inserted at both ends of the gene. PCR conditions were as follows: a hot start at 94 °C for 5 min, 30 cycles of 94 °C for 30 s, 55 °C for 30 s, and 72 °C for 80 s, and a final extension cycle at 72 °C for 10 min. The amplified PCR product was cloned in-frame at the downstream site of the α-factor (signal peptide) in pPIC9K vector, yielding the recombinant plasmid pPIC9K-ReLipA. The recombinant plasmid was linearized by *Sal*I and transformed into *P. pastoris* GS115.

The colonies with multiple copies of the integrated plasmid were screened on MD (minimal dextrose) plates at different geneticin 418 (Life Technologies, Gaithersburg, MD, US) concentrations (0.5–4.0 mg mL^−1^). Positive colonies were selected and inoculated in 5 mL of BMGY medium (buffered minimal glycerol complex medium) at 30 °C with a rotation speed of 200 rpm till the optical density (OD_600_) reached to 2.0–6.0. The *P. pastoris* cells were then harvested by centrifugation and re-suspended in 10 mL of BMMY medium (buffered minimal methanol complex medium) to obtain a final OD_600_ of 1.0. After the medium was continuously cultured in shake flasks for 5 days, methanol was supplemented every 24 h to ensure a final concentration of 0.5 % (*v/v*) to induce the expression. The culture was centrifuged at 12000×*g* for 20 min, and the supernatant was collected and checked for lipase activity.

The selected strain with the highest lipase activity in shake-flask culture was subjected to high cell-density fermentation in a 5-L fermentor at 30 °C according the method described in the *Pichia* Fermentation Guidelines (Version B, 053,002, Invitrogen Inc.). Samples withdrawn at different time intervals during the methanol induction phase were assayed for OD_600_, wet weight of the cells, lipase activity, and protein concentration.

### Enzyme assay and protein determination

Lipase activity was determined by a spectrophotometric assay using *p*-nitrophenyl laurate (*p*NPL) as the substrate. Namely, 50 μL of substrate solution (dissolved in isopropanol with a final concentration of 10 mM) was mixed with 400 μL of 50 mM citrate buffer (pH 6.0), and pre-incubation at 40 °C for 2 min, and then the reaction was initiated by the addition of 50 μL of appropriately diluted enzyme. After incubation at 40 °C for 10 min, the reaction was terminated by adding 500 μL of a moving alkaline copper phosphate suspension. The mixture was centrifuged at 10000×*g* for 3 min, and the absorbance of the supernatant at 410 nm was measured immediately. One unit of enzyme activity was defined as the amount of enzyme liberating 1 μmol of *p*NP per minute under the above conditions. Protein concentration was measured by the method of Lowry et al. [43] using bovine serum albumin (BSA) as the standard.

### Purification of the recombinant lipase

The cell-free crude supernatant was harvested by centrifugation (10000×*g*) at 4 °C for 20 min, and dialyzed against buffer A (20 mM citrate buffer pH 5.0) for 16 h. The dialysate was then loaded onto a SP Sepharose column pre-equilibrated with buffer A. After washing with buffer A till the OD_280_ reached to baseline, the bound proteins were eluted with 0–200 mM NaCl gradient at a flow rate of 1 mL min^−1^. The fractions showing high lipase activity were collected and checked for purity by SDS-PAGE.

### SDS-PAGE and molecular mass determination

The homogeneity and subunit molecular mass of ReLipA were determined by SDS-PAGE as described by Laemmli [44] using 12.5 % separation gel. Protein bands were visualized by staining with Coomassie Brilliant Blue R-250. The low molecular mass calibration kit (Amersham) contained phosphorylase b (97.0 kDa), albumin (66.0 kDa), ovalbumin (45.0 kDa), carbonic anhydrase (30.0 kDa), trypsin inhibitor (20.1 kDa), and α-lactalbumin (14.4 kDa). The native molecular mass of ReLipA was determined by size-exclusion chromatography on a Superdex-75 gel filtration column (1 × 40 cm) which was pre-equilibrated with 20 mM phosphate buffer (pH 7.0) containing 150 mM NaCl at a flow rate of 0.3 mL min^−1^. Standard proteins used were phophorylase b (97.2 kDa), albumin (66.2 kDa), albumin (45.0 kDa), α-chymotrypsinogen A (25.7 kDa), and α-lactalbumin (14.4 kDa).

### Characterization of the recombinant lipase

The optimal pH of the lipase was determined by measuring enzyme’s activity at 40 °C using the following buffers (50 mM): citrate (pH 3.0–6.0), 2-(*N*-morpholino)ethanesulfonic acid (MES) (pH 5.5–6.5), phosphate (pH 6.0–8.0), Tris–HCl (pH 7.5–9.0). To determine pH stability, the enzyme was incubated in different buffers mentioned above at 30 °C for 30 min, and the residual lipase activity was then assayed in 50 mM citrate buffer (pH 6.0). The optimal temperature was determined by measuring the lipase’s activity in 50 mM citrate buffer (pH 6.0) at different temperatures (0–60 °C). Thermal inactivation studies were performed by measuring the residual activity after pre-incubation of the enzyme in 50 mM citrate buffer (pH 6.0) at a temperature range of 30–60 °C for 30 min.

The effect of some surfactants on the enzyme stability was investigated after incubation of the lipase in 50 mM citrate buffer (pH 6.0) at 30 °C for 1 h in the presence of 5 % (*w/v*) of the following detergents: Tween 20, Tween 60, Tween 80, Triton X-100 and SDS. The stability of the lipase against 30 % (*v/v*) of various organic reagents was evaluated by the similar method. The organic solvents included methanol, ethanol, butanol, acetone, acetonitrile, isopropanol, cyclohexane, and heptane.

### Substrate specificity, kinetic parameters, and positional specificity of ReLipA

Substrate specificity of the purified lipase was determined by measuring the enzyme’s activity according to the standard assay using different *p*NP esters (*p*NPA, *p*NPB, *p*NPH, *p*NPC, *p*NPD, *p*NPL, *p*NPM, and *p*NPP), and triglycerides (triacetin, tributyrin, tricaproin, tricaprylin, tricaprin, trilaurin, trimyristin, and tripalmitin) as the substrates. The kinetic parameters of ReLipA toward *p*NPC (C_8_), *p*NPL (C_12_), and *p*NPM (C_14_) were determined by measuring the enzyme’s activity in 50 mM citrate buffer (pH 6.0) at 40 °C for 5 min using different substrate concentrations. The *K*_m_ and *V*_max_ values were calculated by “GraFit” software.

The positional specificity of ReLipA was examined by analyzing the hydrolysis products of triolein using thin layer chromatography (TLC). Briefly, 1 mL of reaction mixture containing 50 mM citrate buffer (pH 6.0), 100 mM triolein, and 50 U of lipase was incubated at 40 °C for 8 h with a shaking speed of 180 rpm. The reaction products were extracted with the same volume of *n*-hexane, and then analyzed by TLC as the method described by Lu et al. [45].

### Synthesis of biodiesel by ReLipA

The reaction mixture including 5 mL of isooctane solvent containing 1.68 g oleic acid, 1098 μL of alcohols (methanol, ethanol, or butanol), and 100 U of the lipase in a 25-mL triangular flask was incubated at 35 °C for 24 h with continuously shaking (150 rpm). The samples were withdrawn at different time intervals, and analyzed by TLC according to the method of Guan et al. [36]. The products were quantified by gas chromatography (Agilent 6890 N) which was equipped with a DB-wax capillary column (30 m × 0. 25 mm × 0. 25 μm) and a flame ionization detector (FID). Nitrogen was used as a carrier gas. Both the injector and detector temperatures were held at 250 °C. The initial column temperature at 180 °C for 3 min was increased to 200 °C by 10 °C min^−1^ and held steady for 3 min, and then increased to a final temperature of 230 °C at a rate of 3 °C min^−1^.

## Abbreviations

BMGY: buffered minimal glycerol complex medium
MES: 2-(*N*-morpholino)ethanesulfonic acid
ORF: open reading frame
*p*NP: *p*-nitrophenol
*p*NPA: *p*-nitrophenyl acetate
*p*NPB: *p*-nitrophenyl butyrate
*p*NPC: *p*-nitrophenyl caprylate
*p*NPD: *p*-nitrophenyl decanoate
*p*NPH: *p*-nitrophenyl hexanoate
*p*NPL: *p*-nitrophenyl laurate
*p*NPM: *p*-nitrophenyl myristate
*p*NPP: *p*-nitrophenyl palmitate
TLC: thin layer chromatography
